# Visuospatial and Sensory Integration Tasks in Patients With Schizophrenia or Schizoaffective Disorder: Relationship to Body Mass Index and Smoking

**DOI:** 10.3389/fpsyt.2018.00473

**Published:** 2018-10-16

**Authors:** Tyler S. Vanderhoof, Tamara V. Gurvits, Julie E. Baker-Nolan, David Borsook, Igor Elman

**Affiliations:** ^1^Boonshoft School of Medicine, Wright State University, Dayton, OH, United States; ^2^Retired, Fort Myers, FL, United States; ^3^Center for Pain and the Brain, Boston Children's Hospital, Massachusetts General Hospital, Harvard Medical School, Boston, MA, United States; ^4^McLean Hospital, Belmont, MA, United States; ^5^Department of Psychiatry, Cooper Medical School, Rowan University, Camden, NJ, United States

**Keywords:** psychosis, metabolism, addiction, visuospatial, nicotine

## Abstract

Neurological soft signs (NSSs) are highly prevalent among patients with schizophrenia, but their pathophysiological significance remains unclear. The present study employed perceptual-motor and visuospatial processing tests that have not yet been attempted in this patient population. Patients with schizophrenia or schizoaffective disorder (*n* = 42) and mentally healthy subjects (*n* = 10) were administered Copy Figure Test, Detection and Recognition of an Object Test and Road Map Test. As compared to controls, schizophrenic and schizoaffective patients displayed significantly poorer ability to copy three-dimensional figures (namely, Necker- and hidden line elimination cubes) and to orient in space on a road-map test; group differences in copying two-dimensional figures and on objects' recognition against a background noise were not apparent. In the schizophrenia/schizoaffective group, more mistakes on the hidden line elimination cube was associated with greater body mass index and greater severity of nicotine dependence measured via the Fagerstrom Test of Nicotine Dependence. The above findings replicate those of prior reports and extend them to the tasks that do not involve motivational and attentional confounds. Furthermore, the present data support the hypothesis that subtle cerebral cortical abnormalities detected with specific NSSs tests may be related to some aspects of metabolic and motivational function in patients with schizophrenia/schizoaffective disorder.

## Introduction

Obesity and smoking are identified by the World Health Organization as leading preventable causes of mortality in the industrialized world^1^. In schizophrenia these risk factors are 2–3-fold more prevalent then in the general population, afflicting over half of the patients (1, 2) thus contributing to the 15–20 years drop in their life expectancy (3, 4). While excessive body weight gain and nicotine consumption could be attributed to a constellation of endocrine, molecular, genetic, demographic, and lifestyle-related factors, the pathophysiological significance of these findings in schizophrenia remains unclear. Identification of their clinical correlates could therefore provide new leads for the development of therapeutic interventions aimed at curbing weight gain and smoking by schizophrenic patients (5, 6).

One of the most consistent findings in clinical studies of schizophrenia is the heightened prevalence of temporally stable (7, 8) markers of neurological compromise namely, neurological soft signs (NSSs) (9, 10). Contrary to the “hard signs,” NSSs are not traceable to a specific brain structure but are rather attributable to wider brain regions and functionally interconnected neuroanatomical systems involved in integrative neurological functions, such as sensory perception, coordination and motor sequencing (11, 12). The ability of different NSSs to explain specific aspects of schizophrenia symptomatology varied and so significant correlation with positive- (13, 14) and negative (15) symptoms or no correlation (16, 17) been reported. The nonspecificity of schizophrenia NSSs findings may be partially attributable to the multidimensionality of the tests employed (18) along with attentional and motivational deficits inherent in schizophrenia neuropsychopathology (19).

In a previous paper, we reported, using easily administered tasks, which impose few cognitive demands and thus not substantially affected by performance confounds (20, 21) that a behavioral addiction, pathological gambling (22), was characterized by constructional apraxia along with impairments in filtrating visual signal from noise and orienting in space (20). Notably, the Copy Figure Test (CFT) for constructional apraxia that is “almost pathognomonic” for brain damage in the presence of grossly abnormal performance on even one out of seven figures (23), was validated across behavioral (20)–and chemical (21) types of addictive behaviors, which also encompass overeating (1) and smoking (24).

Although only one of many legitimate ways that neurological compromise might be operationalized, NSSs' generalized rather than localized nature renders them a relevant investigational target in patients with schizophrenia wherein the list of brain regions that have been found to differ on various comparisons stretches from cerebellum to prefrontal cortex (25, 26). Clinical rationale for this sort of investigation is provided by the use of a legal stimulant (27) e.g., nicotine as a potential form of self-medication for visuospatial abnormalities reflected in NSSs (28, 29). Additionally, some specific visuospatial abnormalities like those uncovered with the CFT, may point to the parietal dysfunction (23) which while been implicated in sensitization to stress (30) and to other motivational targets (31) is relatively unaddressed in schizophrenia and addiction literatures. Moreover, obesity and insulin resistance may impair visuospatial functions (32) driving high sugar- and fat content diets in conjunction with nicotine consumption that provide momentary relief (33–35) but eventually lead to impairments in physiologic mechanisms regulating food intake and impulse control and so contribute to progressive worsening of the clinical condition.

Accordingly, we administered to patients with schizophrenia or schizoaffective disorder visuospatial and sensory integration tasks that were carefully chosen from a comprehensive NSSs assessment battery due to their sensitivity and discriminative ability in addicted- (20, 21) and in other types of psychiatric patients (36) as well as due to convenience of administration as paper-and-pencil tasks. The tasks were comprised of: (a) CFT: copying two- and three-dimensional figures (20, 21); (b) Detection and Recognition of an Object [against a background noise] Test (20) (DROT); and (c) left-right orientation in the form of reading and understanding a simple road map (20). Consistent with prior reports (12) we hypothesized that relative to healthy comparison subjects, patients with schizophrenia or schizoaffective disorder would display compromised performance on the three tests above. In an exploratory fashion, that is to say, in order to formulate hypotheses for future investigation potential relationships between body mass index (BMI), smoking severity and positive/negative schizophrenia symptoms were also assessed.

## Methods

### Subjects

Forty-two olanzapine-treated subjects diagnosed with schizophrenia (*n* = 34) or schizoaffective disorder (*n* = 8) were recruited via advertisement to participate in a double blind placebo-controlled clinical trial the results of which are reported elsewhere (37). Diagnoses were made using the Structured Clinical Interview (38) for Diagnostic and Statistical Manual of Mental Disorders (SCID), Fourth Edition, Text Revision (39) and clinical history via interview with Board-Certified Psychiatrist. Each participant provided written consent after receiving full explanation of the Mclean Hospital IRB-approved procedures. Subjects' inclusion was unrelated to their performance on the study tasks. Subjects were excluded based on diagnosis of dementia, bipolar disorder, major depression, drug/alcohol dependence, or eating disorder. Exclusion criteria also incorporated a potentially confounding medical- (e.g., the presence of a pacemaker, diabetes mellitus, other endocrinopathy, chronic obstructive pulmonary disease, congestive heart failure, hepatitis, hepatic failure, cirrhosis, HIV positive status, end-stage kidney disease, use of opioid agonists or antagonists or use within the past month of drugs with prominent orexigenic, anorexigenic effects) or neurological (e.g., seizure disorder, head trauma, past brain surgery, multiple sclerosis, or Parkinson's disease) condition along with pregnancy or plans to become pregnant. Healthy control subjects (*n* = 10) had no psychiatric history as determined by SCID; their data were previously reported (20).

All patients were stable outpatients with a chronic course of illness and were tested during treatment with a stable dose of olanzapine (mean = 14.0 ± 7.4 mg/day, range: 5–30 mg/day) for a minimum of 2 months. The total scores on the Scale for the Assessment of Negative Symptoms (mean = 41.56 ± 22.08; range: 0–125) (40), on the Positive and Negative Symptoms Scale (mean = 36.0 ± 18.40; range: 30–210) (41) and on the Brief Psychiatric Rating Scale (mean = 39.81 ± 10.07; range: 24–168) (42) were indicative of low to moderate symptom levels.

### Procedures

**Figure 1:**
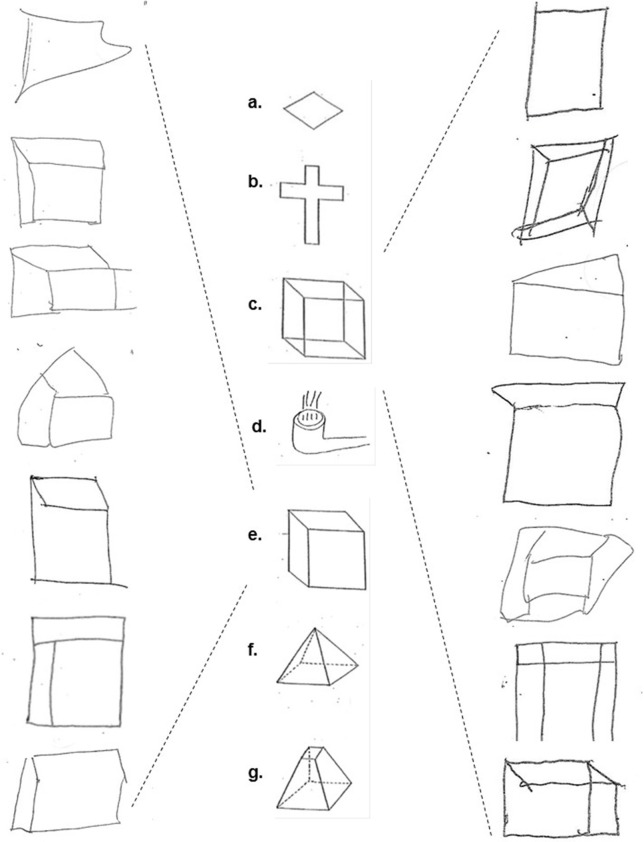
The copied figures: (a) diamond, (b) cross, (c) Necker cube, (d) smoking pipe, (e) hidden line elimination cube, (f) pyramid, and (g) dissected pyramid (middle column); Examples of schizophrenia/schizoaffective subjects' performance on the Necker cube (right column) and hidden line elimination cube figures (left column).

The three tests were administered in sequential order during one session. The CFT comprised seven figures that the participants were instructed to copy exactly as they appeared with a pen and to not attempt to erase any mistakes made (Figure 1). The figures were available for the participants to look at for the full duration of the test, eliminating any reliance on memory.

The DROT consisted of six images of basic household objects, including a key, shovel, pitcher, eyeglasses, hammer, and kettle. The recognition of each object was complicated by the addition of background “noise” which consisted of a field of black dots. The six images were presented in two separate sets. The first set of images were in high density (high difficulty; 35 dots per line) while the second set of the same images were placed in low density (low difficulty; 15 dots per line). Participants were instructed to identify each object and could manipulate the page freely.

In the Road Mad Test (RMT) participants were presented with a map of an imaginary town with a route consisting of 32 intersections. Following a brief practice trial to familiarize the participants to the task, each participant was instructed to mentally drive the route, and to sequentially determine whether they were to turn left or right at each intersection to follow the route. The researcher followed the route with a pencil and marked R or L based on the participant's verbal response at each intersection. Throughout the duration of the test, the map remained in a fixed position in front of the subjects without a possibility to move it.

The three tests were scored by a dually trained Board-Certified psychiatrist and neurologist (TVG) who was blind to the subjects' diagnoses and specifically trained in the administration and scoring of these tests. A four-point scale was used for each figure in the CFT. A score of zero (0) was given for perfect or near perfect reproduction; 1 was given for slight distortion or rotation; 2 was given for moderate distortion or rotation, severe scaling mistakes, or loss of three-dimensionality; and 3 was given for severe distortion or an unrecognizable image. The number of failed identifications or of wrong turns were respectively scored for the DROT and the RMT.

Demographic variables were analyzed by Student's *t*-tests or Fisher's exact tests as appropriate. Because most of the CFT, DROT, and RMT data were ordinal and not normally distributed, they were summarized as both median and mean ± standard deviation (SD). The univariate nonparametric Mann-Whitney U test was used to compare groups. The non-parametric Spearman correlation coefficient was used for correlation analyses. Significance was defined as *p* < 0.05, with more abnormalities predicted in the schizophrenia/schizoaffective group.

## Results

Table 1 presents demographic data for the two groups. There were no significant differences between schizophrenic/schizoaffective and control subjects, respectively, for age, gender and education. On average, schizophrenic/schizoaffective subjects were obese (BMI = 30.8 ± 5.2 vs. 28.01 ± 2.01 kg/m^2^ for healthy controls) and endorsed 4.6 ± 2.5 out of 17 items on the developmental history questionnaire (comprised of the following items: born prematurely, trauma in delivery, delay walking, delay talking, attention deficits, hyperactivity, enuresis, problem reading, problem writing, problem with math, repeated grades, head Injury, seizures, positive family history of developmental disorder, physical abuse, sexual abuse and tutoring or special classes); among schizophrenic/schizoaffective subjects 33 reported smoking (Fagerstrom test for nicotine dependence score = 3.2 ± 2.9; range: 0–10); all healthy control subjects were non-smokers.

**Table 1:** Demographic characteristics (means ± SDs or ratios) of study participants.

**Variable**	**PG (*n* = 42)**	**Control (*n* = 10)**	***T*****-test (df** = **50)**	
			***t***	***p***
Age (year)	44.9 ± 10.6	43.6 ± 14.2	0.33	0.20
Education (year)	13.5 ± 3.2	15.1 ± 1.4	−1.56	0.12
			Fisher's exact test (df = 1)	
Gender (M/F)	26/16	5/5		0.50

Table 2 presents the group medians and means for each figure separately, DROT and RMT along with results of the Mann-Whitney U Test pairwise group comparisons. It may be seen that there was no significant group effect on copying diamond, cross, smoking pipe, pyramid and dissected pyramid figures along with high and low difficulty DROT versions. For Necker- and hidden elimination cubes and RMT, there were significant differences between schizophrenic/schizoaffective subjects (worse performance) vs. healthy subjects (Figure 1).

**Table 2:** Group medians and mean (±SDs) for the performance indices on the copy figure, detection and recognition of an object and the road map tests.

**Task**	**Schizophrenia (*n* = 42)**	**Control (*n* = 10)**	**Mann-Whitney** ***U*** **Test**	
	**Median mean ±SD**	**Median mean ±SD**	***U***	***p***
CFT (score; 0–3)				
Diamond	0	0	172	0.29
	0.4 ± 0.5	0.2 ± 0.4		
Cross	0	0	199	0.78
	0.6 ± 0.8	0.4 ± 0.5		
**Necker cube**	**2**	**0**	**119**	**0.03**
	**1.6** ±**1.3**	**0.6** ±**0.8**		
Smoking pipe	0	0	144	0.09
	1.0 ± 1.2	0.2 ± 0.4		
**Hidden elimination cube**	**1**	**0**	**115**	**0.02**
	**1.4** ±**1.1**	**5 0.5** ±**0.5**		
Pyramid	1	0	145	0.12
	1.2 ± 1.2	5 0.5 ± 0.5		
Dissected pyramid	1	0	143	0.10
	1.8 ± 1.2	0.6 ± 1.0		
DROT error (#)				
High noise	3	3	142	0.12
	3.4 ± 1.5	2.8 ± 0.9		
Low noise	2	1	147	0.25
	2.1 ± 1.7	1.4 ± 1.3		
**RMT error (#)**	**7**	**1**	**71**	**0.001**
	**6.9** ±**5.4**	**1.0** ±**1.2**		

**Figure 2:**
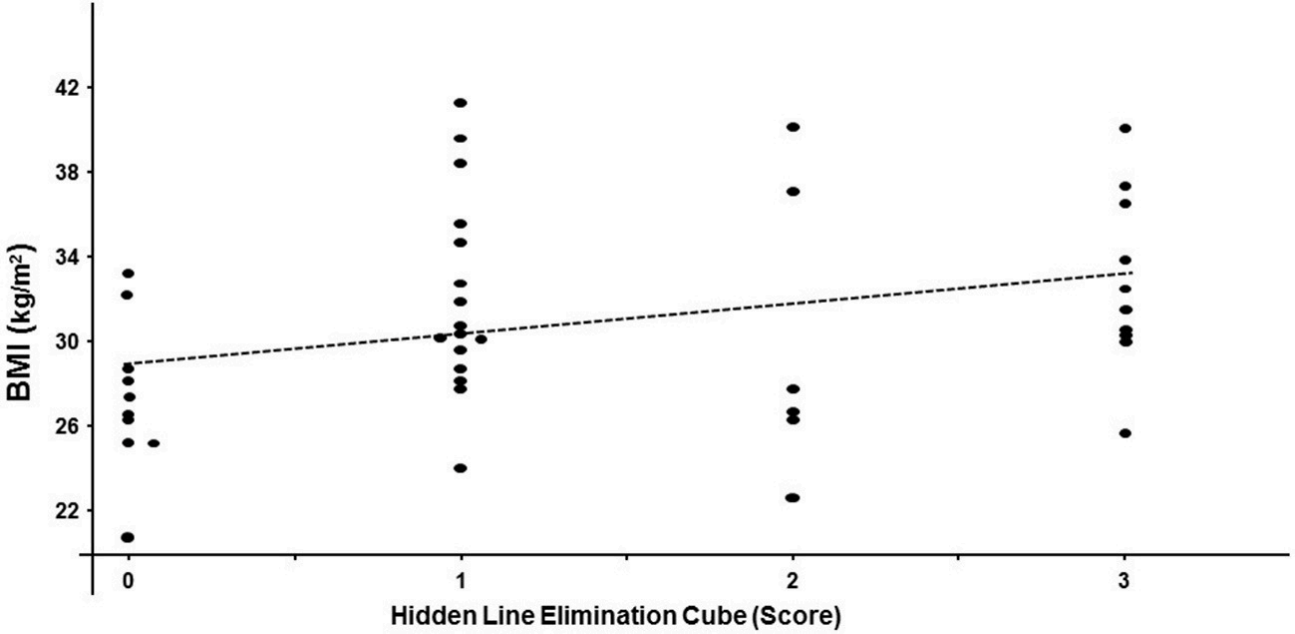
Scatterplot relating individual hidden elimination cube scores and BMI values in patients with schizophrenia/schizoaffective disorder using Spearman coefficient (r_s_ = 0.32; df = 40; *p* = 0.04).

**Figure 3:**
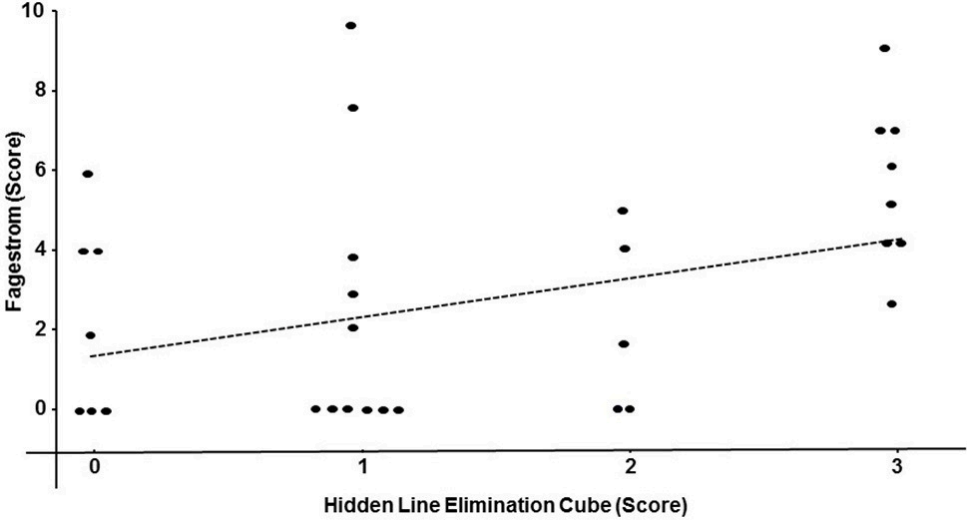
Scatterplot relating individual hidden elimination cube- and the Fagerstrom Test for Nicotine Dependence -scores in patients with schizophrenia/schizoaffective disorder using Spearman coefficient (r_s_ = 0.35; df = 31; *p* = 0.04).

Performance on the hidden elimination cube (but not on Necker cube or RMT; *p* > 0.13) correlated significantly with BMI (r_s_ = 0.32; df = 40; *p* = 0.04; Figure 2) and with the Fagerstrom Test for Nicotine Dependence score (r_s_ = 0.35; df = 31; *p* = 0.04; Figure 3). There was a trend toward correlation between performance on the hidden elimination cube and a number of positive developmental items (*p* = 0.056). No relationships were observed between SANS, PANSS and BPRS scores on those for the Necker- and hidden elimination cubes and RMT (*p* > 0.16).

## Discussion

To our knowledge, this is the first study to integrate NSSs related to perceptual-motor and visuospatial processing NSSs using CFT, DROT and RMT in schizophrenic/schizoaffective patients. The major findings are worse performance on the copying of some tri-dimensional (i.e., Necker- and hidden line elimination cubes), but not two-dimensional figures and impaired left-right orientation in the patients' group. The constructional apraxia uncovered by the CFT was not secondary to visual agnosia as evidenced by the DROT findings. Although the employed tasks did not constitute real life challenges, our findings may have clinical significance in patients with schizophrenia because difficulties with perceptual (43) and spatial integration along with other neurocognitive deficits may be a potential explanation for heightened rates of car accidents (44) in schizophrenics considering that proper copying cube ability (45) and left-right orientation (46, 47) are important prerequisites for safe driving. Such deficits may relate to lateralized brain dysfunction in these patients (48) that has some basis in altered brain connectivity (49).

Our data are consistent with a large body of literature reporting that patients with schizophrenia have a greater prevalence of NSSs, including various types of apraxia (50, 51), left-right disorientation (52), than other psychodiagnostic categories (9, 12) reaching 100% in some of the series when non-restrictive definitions of the NSSs are used (12, 16, 53). Although there were substantial methodological similarities between the prior studies with regard to the patient population and to the examination of NSSs, the important novel aspect here was the tasks *per se*. Thus, this independent replication strongly supports the validity of schizophrenia-related constructional and visuospatial impairments as defined by the easily administrated paper-and-pencil tasks (20, 21) selected for their discriminative ability in patients with chemical (21) and behavioral (20) addictions.

Conspicuous clinical and neuropathological similarities indeed exist between schizophrenia and various types of addictions (1). Clinically, drug-induced psychosis may be indistinguishable from schizophrenia symptoms (54, 55), whereas predisposition for addictive behaviors is ingrained in schizophrenia neuropathology owing to alterations that are analogous to those of substance abuse even in absence of prior drug exposure (1, 56). On the other hand, some schizophrenia neuropsychopathology may not overlap with addiction as dopaminergic surges associated with these types of syndromes are not entirely of the same magnitude and on the same time scale (57). Accordingly, there are no parallels in the schizophrenia literature to the well-recognized symptoms of craving, tolerance and withdrawal by patients with addictive disorders. Other potential differences include perceptual advantage in the assessment of three-dimensional objects (i.e., stereopsis) particularly by psychotic and disorganized schizophrenic patients (58, 59) that may be attributed to improved dopamine-related (60) prediction-error signaling updating expectations about future events based on discrepancies between past expectations and experiences (59). Such a seemingly paradoxical state may be adaptive from a phylogenetic perspective as it advances coping mechanisms not only during psychotic outbreaks, but also during sleep deprivation and withdrawal from alcohol (61).

In comparison to our previously reported data from patients with cocaine dependence (21) and with pathological gambling (20), the present sample of schizophrenic/schizoaffective patients were found to have seemingly better scores on drawing of the tri-dimensional figures in conjunction with a better DROT performance (20). Importantly, scores on the hidden line elimination cube probing stereopsis c.f., Necker cube, necessitating visuospatial ability to shift attention between two equally plausible figural spatial representations (62), correlated with the BMI and the Fagerstrom Test of Nicotine Dependence. While some of the above findings might be accounted by differences in the respective illness' severity, which complicates direct comparisons between the schizophrenic/schizoaffective and addicted groups, the presented data call for further research aimed at understanding the distinctive features of constructional apraxia revealed by copying the hidden line elimination cube *vis-à-vis* copying other figures in conjunction with the DROT and RMT and their potential role in metabolic and addictive disturbances associated with schizophrenia/schizoaffective disorder.

The low PANSS scores are consistent with clinical stability and a lack of significant correlation may be due to the minimal variance i.e., “floor effect.” Also, as it is the case for all cross-sectional studies, the limitation of the present design is its inability to resolve the origin of elevated NSSs in the patient group. The possibility that NSSs are preexisting vulnerability markers is supported by their presence in mentally healthy relatives of schizophrenic patients (63, 64). The tentative correlation between performance on the hidden elimination cube and a number of positive developmental items would further support the preexisting and even inheritable trait-like NSSs' nature. Schizophrenia does have a robust genetic component ranging from 50 to 60%^2^. Greater premorbid hyperactivity, impulsivity and left/right confusion have been found in schizophrenic patients (65). Nonetheless, another possible origin of NSSs in schizophrenic/schizoaffective patients is that they are acquired, e.g., they are a consequence of psychotic episodes with resultant phasic dopaminergic bombardment and consequent brain alterations. Prospective clinical studies are warranted to address the NSSs' state vs. trait features (66) and their relative chronology (e.g., predate or follow the development of psychotic symptoms).

The exploratory and correlational design of this study does not yet prove direct interaction of constructional ability with BMI or smoking. Likewise, it cannot be determined from our results whether visuoperceptual deficits and obesity and/or smoking both are function of a third factor (e.g., subtle brain damage) or result from each other. Accordingly, these findings may provide a foundation for further, more rigorously designed projects. Such projects may include prospective design with a longitudinal NSSs assessment (including visuospatial and sensory integration tasks) in relation to food and nicotine intake, metabolic status and clinical course of the illness.

This pilot study has several additional limitations, including the small sample size, explorative methodology, and the sole reliance on the visuospatial and sensory integration tasks selected from the 45-item battery of NSSs tests available for neuropsychiatric research (36). Consequently, schizophrenia findings may not necessarily be limited to the deficits uncovered by the employed tasks and could generalize to other neurological and/or cognitive impairments. Future studies employing more comprehensive assessments e.g., motor functions that are critical components of NSSs, such as motor coordination and sequencing may shed additional light on the complex interrelationship of schizophrenia with visuospatial and sensory Integration, BMI and smoking.

Nonetheless, the presented findings add to the substantial body of evidence implicating NSSs in the course of schizophrenia/schizoaffective disorder by suggesting that the relationship is relatively specific to the traits defined by copying Necker- and hidden line elimination cube as well as the left-right orientation and do not generalize to other forms of constructional apraxia or visual agnosia. More research is needed to determine which aspects of NSSs are most strongly associated with schizophrenic neuropsychopathology and to compare them to other syndromes with dopaminergic etiology, such as chemical and behavioral addictions as well as depression and post-traumatic stress disorder (67). Furthermore, the relationship of NSSs to disease state and treatment effects are not known and require further elucidation. The role of NSSs as a clinical tool in evaluating schizophrenia symptoms requires further validation in conjunction with definition of sensitivity and specificity.

## Author contributions

IE and TG conceived and designed the experiments. IE performed the experiments. IE, JB-N, and TV analyzed the data. TG, DB, and JB-N contributed reagents, materials, and analysis tools. TV, TG, IE, JB-N, and DB wrote the paper.

### Conflict of interest statement

The authors declare that the research was conducted in the absence of any commercial or financial relationships that could be construed as a potential conflict of interest.

## Footnotes

^1^World Health Organization. mortality and burden of disease attributable to selected major risks. WHO; 2009. Global health risks.http://www.who.int/healthinfo/global_burden_disease/GlobalHealthRisks_report_full.pdf.

^2^Heredity and the Genetics of Schizophrenia: http://www.schizophrenia.com/research/hereditygen.htm.
